# Hepatic arterial infusion chemotherapy vs transcatheter arterial embolization for patients with huge unresectable hepatocellular carcinoma

**DOI:** 10.1097/MD.0000000000021489

**Published:** 2020-08-07

**Authors:** Wei-Lun Tsai, Wei-Chi Sun, Wen-Chi Chen, Chia-Ling Chiang, Huey-Shyan Lin, Huei-Lung Liang, Jin-Shiung Cheng

**Affiliations:** a Division of Gastroenterology and Hepatology, Department of Internal Medicine, Kaohsiung Veterans General Hospital, Kaohsiung; b Shool of Medicine, National Yang-Ming University, Taipei; c Department of Radiology, Kaohsiung Veterans General Hospital; d School of Nursing, Fooyin University, Kaohsiung, Taiwan.

**Keywords:** chemotherapy, hepatocellular carcinoma, huge, unresectable

## Abstract

For the treatment of huge unresectable hepatocellular carcinoma (HCC), transcatheter arterial chemoembolization (TACE) or transcatheter arterial embolization (TAE) generally had poor effects and high complication rates. Our previous study found that Hepatic arterial infusion chemotherapy (HAIC) is a safe procedure and provides better survival than symptomatic treatment for the patients with huge unresectable HCC. The aim of the study is to compare the effect of HAIC vs TAE in patients with huge unresectable HCC.

Since 2000 to 2005, patients with huge (size > 8 cm) unresectable HCC were enrolled. Twenty-six patients received HAIC and 25 patients received TAE. Each patient in the HAIC group received 2.5 + 1.4 (range: 1–6) courses of HAIC and in the TAE group received 1.8 + 1.2 (range: 1–5) courses of TAE. Baseline characteristics and survival were compared between the HAIC and TAE group.

The HAIC group and the TAE group were similar in baseline characteristics and tumor stages. The overall survival rates at 1 and 2 years were 42% and 31% in the HAIC group and 28% and 24% in the TAE group. The patients in the HAIC group had higher overall survival than the TAE group (*P* = .077). Cox-regression multivariate analysis revealed that HAIC is the significant factor associated with overall survival (relative risk: 0.461, 95% confidence interval: 0.218–0.852, *P* = .027). No patients died of the complications of HAIC but three patients (12%) died of the complications of TAE.

In conclusion, HAIC is a safe procedure and provides better survival than TAE for patients with huge unresectable HCCs.

## 1 Introduction

Hepatocellular carcinoma (HCC) is the fifth most common cancer in the world and ranked the 2nd cause of cancer death in Taiwan.^[1,2]^ Although routine screening for high risk patients, huge HCCs with size of more than 8 cm are occasionally seen.^[3]^ Surgical resection is considered to be the standard curative therapy for huge HCC in patients with good liver reserve.^[2,4–8]^ According to the study from Kaohsiung Veterans General Hospital, Mok et al found that the advantage of hepatic resection in patients with huge HCC is marginal as compared with multimodality treatment including transcatheter arterial embolizatoin (TAE) or hepatic arterial infusion chemotherapy (HAIC).^[9]^ However huge HCC often presented with poor liver reserve, with increased frequency of intrahepatic metastasis and vascular invasion, which made surgical resection not suitable. So transcatheter arterial embolization/chemoembolization (TAE/TACE) has been considered as the choice for the palliative treatment of huge unresectable HCC.^10^ However previous studies found that TACE for huge HCC had poor effect, and TACE related mortality rate of 6.5% to 20% has been reported.^[10,11]^ HAIC is another option for the palliative treatment for inoperable advanced HCC.^[12–15]^ In our previous study, HAIC with cisplatin, mitomycin C, leucovorin and 5-FU for advanced unresectable HCC had tumor response rate of 28.3% and only one patient died due to the complication of HAIC during 211 courses of treatments.^[16]^ From another recent study from our hospital, HAIC for advanced HCC had overall response rate of 20%.^[17]^ Our recent study also found that HAIC provided survival benefit over symptomatic treatment in patients with huge unresectable HCC and no patients died of the immediate complications of HAIC.^[18]^ So HAIC seemed to be an effective and safe method for the treatment of huge unresectable HCC. But the effect of HAIC vs TAE for the treatment of huge unresectable HCC remained unclear. The aim of the study is to investigate the effect of HAIC vs TAE for the treatment of huge unresectable HCC.

## 2 Materials and methods

### 2.1 Patients

From January 2000 to December 2005, consecutive eligible patients with hepatocellular carcinoma (HCC) were enrolled in this study. HCC was diagnosed by pathology or elevation of alpha-fetoprotein (AFP) level above 400 ng/ml along with at least two different imaging techniques including computed tomography (CT) or magnetic resonance imaging (MRI). All patients met the following criteria:

(A) tumor of 8 cm or more in diameter,
(B) patients who were not suitable for operation,
(C) portal vein is patent
(D) platelet counts > 50000/cumm,
(E) prothrombin time INR < 1.5.
(F) white cell counts > 2500/cumm, and
(G) Child A or B liver reserve. Patients with a previous history of treatment for HCC, or distant metastasis were excluded.

**Figure 1:**
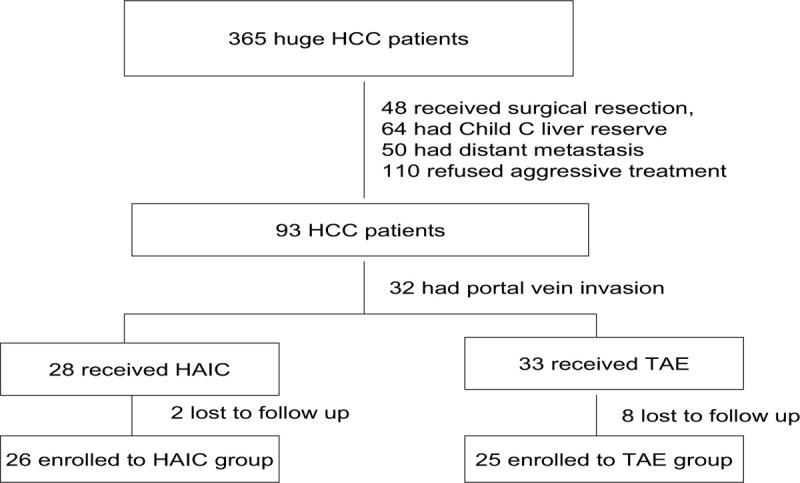
Flowchart summarizes patient inclusion.

From 2000 to 2005, 365 consecutive patients first diagnosed with huge HCC defined as tumor size greater than or equal to 8 cm were admitted to Kaohsiung Veterans General Hospital. Among the 365 patients, 272 were excluded (48 received surgical resection, 64 had Child C liver reserve and 50 had distant metastasis, and 110 refused aggressive treatment). Thirty-two patients who had portal vein invasion were excluded. Among the 28 patients who received HAIC, 2 were lost to follow up and 26 patients were enrolled in the HAIC group. Among the 33 patients who received TAE, 8 patients were lost to follow up, so 25 patients were enrolled in the TAE group (Fig. 1). The choice of either HAIC or TAE was determined in a somewhat random manner by the operator and was not influenced by the clinical condition of the patients.

### 2.2 Hepatic arterial infusion chemotherapy (HAIC)

The left subclavian artery was cannulated with a catheter and the tip of the catheter was placed in the proper hepatic artery under fluoroscopic guidance before each course of chemotherapy.^[13]^ The main trunk of the gastroduodenal artery was occluded by metallic coil routinely. Continuous infusion of 5000 units (5cc) heparin solution daily was filled in the catheter for prevention of occlusion by thrombosis. Each course of treatment was 5 days. Cisplatin (10 mg/m^2^) and mitomycin-C (2 mg/m^2^) were dissolved in 50 ml isotonic sodium chloride solution which was infused for 20 to 30 minutes each time and continued for 5 days. In addition, 100 mg/ m^2^ of 5-fluorouracil (5-FU), dissolved in 250 ml of isotonic sodium chloride solution was administered for 24 hours by infusion pump for 5 days. Leucovorin (15 mg/m^2^) was given daily to improve the efficacy of 5-FU during HAIC. The interval between 2 courses of treatment was 3 to 4 weeks. Each patient received at least one session of treatment. Three-phase computed tomography (CT) scan of liver was done after every 2 courses of treatment. Termination of treatment when patients received 6 courses of treatment or until clinical conditions of the patients were not suitable for another course of HAIC.

### 2.3 Transcatheter arterial embolization (TAE)

TAE was performed through selective hepatic arterial catheterization. Whenever possible, the arteries that supply the tumor were catheterized superselectively and 5 to 15 ml of lipiodol was injected, followed by embolization with small gelfoam pellets of 1x1 mm in size. CT scan of liver was performed 2 to 3 months after TAE and further TAE was performed every 2 to 3 months if viable or recurrent tumors were found and patient had suitable liver reserve and no contraindication for TAE. All patients were followed by CT or MRI of liver and AFP every 3 months.

### 2.4 Follow-up

All patients in the HAIC group who completed total 6 courses of chemotherapy or not suitable for further chemotherapy or patients in the TAE group who were not suitable for further TAE received follow-up with liver function test, AFP, sonography, CT scan or MRI of liver every 3 months.

### 2.5 Statistical analysis

The data were expressed as mean + standard deviation. Categorical variables were compared with the *X*^2^ test or Fisher exact test when appropriate and continuous variables were compared with the Mann-Whitney test. Overall survival was estimated using the Kaplan–Meier method and the difference was determined by the log-rank test. Univariate and multivariate analysis were performed using Cox's regression model with proportional hazards. A *P* value of less than .05 was considered as statistically significant.

The study was approved by the Kaohsiung Veterans General Hospital Institutional Review Board. This was a retrospective study without intervention or obtaining clinical specimens and all the data were analyzed anonymously, so informed consent was waived. The waiving of informed consent was approved by the Institutional Review Board of Kaohsiung Veterans General Hospital.

## 3 Results

The baseline characteristics of patients in the HAIC group and the symptomatic treatment group were similar in age, sex, tumor size, tumor number, ALT level, albumin level, bilirubin level, presence of ascites, Child's classification, Okuda stage, CLIP stage, BCLC substage and AJCC stage (Table 1).^[19,20]^

**Table 1:**
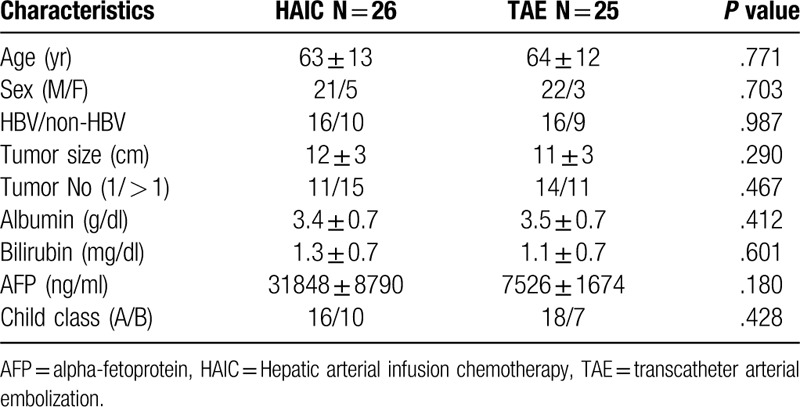
Baseline characteristics of the patients in the HAIC or TAE group.

Total 64 courses of HAIC were performed for the 26 patients in the HAIC group. Each patient received 2.5 + 1.4 (range: 1–6) courses of HAIC. No patients died of the immediate complications of HAIC. One patients developed bacteremia during HAIC and were treated successfully by antibiotics. Total 45 courses of TAE were performed for the 25 patients in the TAE group. Each patient received 1.8 + 1.2 (range: 1–5) courses of TAE. Three patients (12%) died of the immediate complications of TAE (one died of tumor rupture and two died of liver failure). One patient developed liver abscess after TAE and resolved after pig-tail drainage and antibiotics treatment.

**Figure 2:**
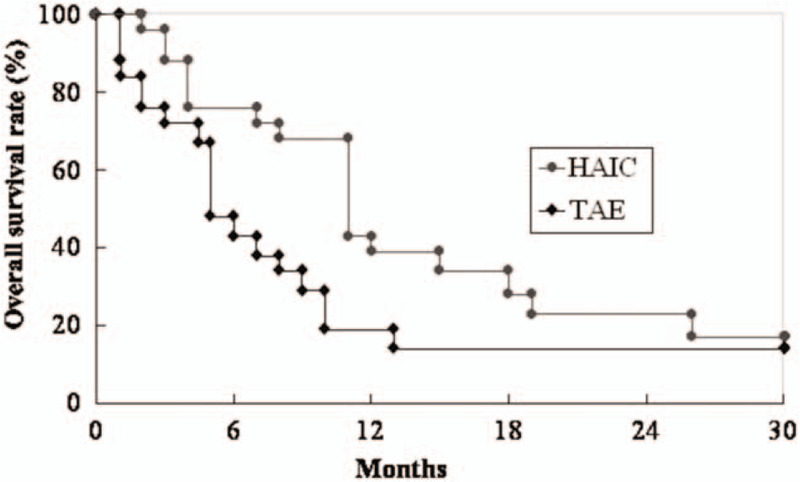
Comparison of the overall survival rate between the HAIC and TAE group. The patients in the HAIC group had higher overall survival than the TAE group (*P* = .077).

Mean follow-up time was 8.3 + 11 months (range: 1–45 months). The overall survival rates at one and two years were 42% and 31% in the HAIC group and 28% and 24% in the TAE group. The patients in the HAIC group had higher overall survival than the TAE group with borderline statistical significance (*P* = .077) (Fig. 2). Cox-regression multivariate analysis revealed the significant factor associated with overall survival were HAIC (relative risk: 0.461, 95% confidence interval: 0.218–0.852, *P* = .027) and AFP level (relative risk: 1.000, 95% confidence interval: 1.000–1.000, *P* = .005) (Table 2).

**Table 2:**
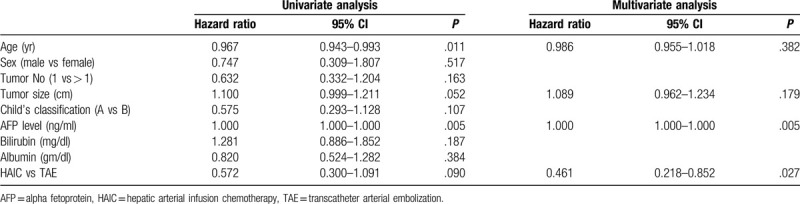
Factors associated with overall mortality in the HAIC or TAE group.

## 4 Discussion

Surgical resection is the treatment of choice for patients with huge HCC and well-preserved liver function.^[5–8]^ However, only a small proportion of patients with huge HCC can fit the criteria for surgical resection. But patients with huge HCC often had a higher prevalence of extracapsular tumor invasion into liver parenchyma, more frequent intrahepatic metastasis and worse survival than those with smaller tumors.^[21–23]^ Our recent study also found that HAIC provided survival benefit over symptomatic treatment in patients with huge unresectable HCC and no patients died of the immediate complications of HAIC.^[18]^ There remained much controversies regarding the treatment for huge unresectable HCC.

Although TAE/TACE has been considered as the choice for the palliative treatment of huge unresectable HCC, severe liver injury after TAE/TACE was anticipated in patients with huge HCC and treatment related mortality rate as high as 20% has been reported.^[11]^ Large tumor size was also found to be a poor prognostic factor in patients undergoing TACE.^[11,24]^ In our hospital, HAIC has been found to be effective and safe for the treatment of advanced or huge unresectable HCC.^[16–18]^ Besides, according to the study by Yamasaki et al, tumor size was not a prognostic factor that influenced the outcome of HAIC for patients with advanced HCC.^[12]^ Studies to compare the treatment outcome of HAIC vs TAE for huge unresectable HCC have never been reported before. This is the first study that compared the treatment outcome of HAIC and TAE in patients with huge unresectable HCC and we found that HAIC is the independent factor associated with overall survival.

HAIC was performed every 3 to 4 weeks and treatment was terminated when patients received 6 courses of treatment or until clinical conditions of the patients were not suitable for another course of HAIC, but TAE was performed every 2 to 3 months if viable or recurrent tumors were found and patient had suitable liver reserve and no contraindication for TAE; Longer interval between each TAE and poor tumor response and deterioration of liver reserve may explain only 1.8 courses of TAE was performed.

During the 64 courses of HAIC, most patients tolerated the procedure well and no patients died of the immediate complications of HAIC. However, the mortality rate related to TAE in this study was 12%. So HAIC may be a more safe treatment procedure for the treatment of huge unresectable HCC.

From a previous randomized controlled study in our hospital, TAE compared with TACE had similar effect for the treatment of HCC.^[25]^ Several other studies that directly compared TAE and TACE did not provide evidence of survival advantages favoring TACE.^[26–29]^ From the results of these studies, TACE did not have significant survival benefit over TAE for the treatment of HCC. So TAE instead of TACE was performed in this study.

Sorafenib has been developed and is recommended for the treatment of advanced HCC.^[30,31]^ But the effect of sorafanib for HCC is not satisfactory and actually the response rate of sorafenib is low.^[32]^ Effects of sorafenib in patients with huge unresectable HCC is unclear. Besides, sorafenib is limited by a high cost and many patients cannot afford to receive the treatment, so HAIC provided a good treatment option for patients with huge unresectable HCC.

This study has several limitations. This is not a randomized controlled study, and selection bias may be possible in this study. But the baseline characteristics including age, sex, liver reserve, tumor stages are similar between the 2 groups of patients. Although the case numbers in this study are small, using Cox regression multivariate analysis, we found that the HAIC group has survival benefit over patients who received TAE. Further randomized controlled studies that enrolled more patients are required to compare the outcome of HAIC vs TAE/TACE for huge unresectable HCC.

In conclusion, HAIC is a safe procedure and provided better survival than TAE for patients with huge unresectable HCCs.

## Author contributions

**Conceptualization:** Wei-Lun Tsai, Huei-Lung Liang, Jin-Shiung Cheng.

**Data curation:** Wei-Chi Sun, Chia-Ling Chiang, Huey-Shyan Lin, Huei-Lung Liang.

**Formal analysis:** Wei-Lun Tsai, Huey-Shyan Lin, Huei-Lung Liang, Jin-Shiung Cheng.

**Investigation:** Wei-Lun Tsai, Wei-Chi Sun, Chia-Ling Chiang, Huei-Lung Liang, Jin-Shiung Cheng.

**Methodology:** Wei-Lun Tsai, Huei-Lung Liang, Jin-Shiung Cheng.

**Resources:** Wei-Lun Tsai, Wei-Chi Sun, Chia-Ling Chiang.

**Software:** Huey-Shyan Lin.

**Supervision:** Wei-Lun Tsai.

**Writing – original draft:** Wei-Lun Tsai.

**Writing – review & editing:** Huei-Lung Liang, Jin-Shiung Cheng.
